# A method for the analysis of the oligomerization profile of the Huntington’s disease-associated, aggregation-prone mutant huntingtin protein by isopycnic ultracentrifugation

**DOI:** 10.3389/fmolb.2024.1420691

**Published:** 2024-06-27

**Authors:** Raffaella Bonavita, Rosaria Di Martino, Giuseppe Cortone, Antonello Prodomo, Mariagrazia Di Gennaro, Gianluca Scerra, Valentino Panico, Silvia Nuzzo, Marco Salvatore, Sarah V. Williams, Fulvia Vitale, Maria Gabriella Caporaso, Massimo D’Agostino, Francesca M. Pisani, Angeleen Fleming, Maurizio Renna

**Affiliations:** ^1^ Department of Molecular Medicine and Medical Biotechnologies, University of Naples “Federico II”, Naples, Italy; ^2^ Institute of Biochemistry and Cell Biology, National Research Council, Naples, Italy; ^3^ Institute for Endocrinology and Experimental Oncology “G. Salvatore”, National Research Council, Naples, Italy; ^4^ IRCCS SYNLAB SDN, Naples, Italy; ^5^ Department of Physiology, Development and Neuroscience, University of Cambridge, Cambridge, United Kingdom

**Keywords:** protein oligomerization and aggregation, sedimentation profile, cell fractionation, differential ultracentrifugation, Huntington’s disease, HTT

## Abstract

Conformational diseases, such as Alzheimer’s, Parkinson’s and Huntington’s diseases as well as ataxias and fronto-temporal disorders, are part of common class of neurological disorders characterised by the aggregation and progressive accumulation of mutant proteins which display aberrant conformation. In particular, Huntington’s disease (HD) is caused by mutations leading to an abnormal expansion in the polyglutamine (poly-Q) tract of the huntingtin protein (HTT), leading to the formation of inclusion bodies in neurons of affected patients. Furthermore, recent experimental evidence is challenging the conventional view of the disease by revealing the ability of mutant HTT to be transferred between cells by means of extracellular vesicles (EVs), allowing the mutant protein to seed oligomers involving both the mutant and wild type forms of the protein. There is still no successful strategy to treat HD. In addition, the current understanding of the biological processes leading to the oligomerization and aggregation of proteins bearing the poly-Q tract has been derived from studies conducted on isolated poly-Q monomers and oligomers, whose structural properties are still unclear and often inconsistent. Here we describe a standardised biochemical approach to analyse by isopycnic ultracentrifugation the oligomerization of the N-terminal fragment of mutant HTT. The dynamic range of our method allows one to detect large and heterogeneous HTT complexes. Hence, it could be harnessed for the identification of novel molecular determinants responsible for the aggregation and the prion-like spreading properties of HTT in the context of HD. Equally, it provides a tool to test novel small molecules or bioactive compounds designed to inhibit the aggregation of mutant HTT.

## 1 Introduction

### 1.1 Protein aggregation and formation of fibrillary tangles in neurodegenerative diseases

Proteinopathies, such as Alzheimer’s (AD), Parkinson’s (PD) and Huntington’s diseases (HD), as well as ataxias and fronto-temporal disorders (FTDs), are part of common class of clinically relevant conditions characterized by the aggregation and progressive accumulation of mutant proteins bearing aggregation-prone, aberrant conformations. Neurodegenerative diseases have many common features, such as their late-onset and chronic nature, increasing prevalence with age, progressive loss of neurons in specific areas of the brain, damage to the synaptic connections and selective loss of cerebral matter (Ross and Poirier, 2004; Bates et al., 2015; Menzies et al., 2017; Jucker and Walker, 2018). In addition, there are common molecular changes that occur within neurons; namely, the progressive accumulation of misfolded protein aggregates in well-ordered structures called amyloid fibrils (Ross and Poirier, 2004; Bäuerlein et al., 2017; Menzies et al., 2017; Jucker and Walker, 2018; Pandey et al., 2018). The pathogenic proteins most commonly implicated in the accumulation of “misfolded” brain aggregates include, among the others, tau and β-amyloid in AD, α-synuclein in PD, huntingtin in HD and FUS, ZNF and TDP-43 in amyotrophic lateral sclerosis (ALS) and Fronto-Temporal Dementia (FTDs) (Jucker and Walker, 2018). These proteins misfold from their native conformations, generating structures rich in intermolecular β-sheets, whose size can range from small oligomeric complexes up to considerably large and insoluble fibrillary aggregates. Amyloid fibrils are large aggregates, 100–200 Å in diameter, characterized from the geometrical standpoint, by a highly ordered and compact arrangement of both intra- and intermolecular β-sheets, displaying a parallel orientation to the longitudinal axis of the fibrils, in a structure known as cross-β (Ross and Poirier, 2004; Menzies et al., 2017; Jucker and Walker, 2018; Pandey et al., 2018). In particular, HD is caused by mutations leading to an abnormal expansion in the polyglutamine (poly-Q) tract of the huntingtin protein (HTT), leading to the formation of inclusion bodies in specific areas of the brain of affected patients. (Davies et al., 1997; Walker, 2007; Bates et al., 2015; Bäuerlein et al., 2017; Pandey et al., 2018; Tao et al., 2019). The severity of the disease correlates with the poly-Q repeat length and arises only in subjects where the poly-Q region comprises 36 CAG repeats or more (Rubinsztein et al., 1996; Kar et al., 2011; Chen and Wolynes, 2017). Despite huge efforts, the molecular mechanisms underlaying the pathogenesis of HD have not been completely elucidated, but the general consensus is that the poly-Q expansion encoded by the first exon of *HTT* is susceptible to atypical folding behaviours, which can elicit a combination of both loss and gain-of-function effects, which in turn affect normal neuronal function and ultimately impact their fitness and survival (Mangiarini et al., 1996; Bates et al., 2015; Bäuerlein et al., 2017; Pandey et al., 2018; Tao et al., 2019). Finally, it is worth noting from the disease model point of view that over-expression of only the first exon of the *HTT* gene is sufficient to phenocopy most of the HD pathogenesis and toxicity both in cell-based and animal model systems (DiFiglia et al., 1995; Gunawardena et al., 2003; Gauthier et al., 2004; Lee et al., 2004; Menzies et al., 2017; Pandey et al., 2018; Masnata et al., 2019; Tao et al., 2019).

To date, to describe the biophysical/biochemical processes leading to the formation of these protein aggregates, reference has been made to the so called “seeding-nucleation model” (as reported by Jarrett and Lansbury, 1993). According to this model, the process is characterised by two phases: a nucleation phase, where stable polymeric nuclei are generated (sometimes referred to as “seeds”); and an elongation phase, in which these nuclei grow rapidly by incorporating the monomeric protein. Large polymers can be fragmented to generate further nuclei that will propagate the reaction. Furthermore, according to the aforementioned model, the preformed nuclei can significantly enhance and facilitate the aggregation process, also recruiting the soluble wild-type protein into the newly constituted, expanding protein aggregate. From a biophysical point of view, protein misfolding and aggregation are multi-step processes, which involves the sequential rearrangement of the protein structure into a series of β-strands, stabilized by H-bonds and characterised by the progressive acquisition and stabilisation of hydrophobic interactions. They have adhesive ends capable of attracting folded or partially folded proteins, causing their misfolding and subsequent insertion into the cross-β6 polymeric structure (Jarrett and Lansbury, 1993).

### 1.2 Huntingtin: structure, role and pathogenesis in HD

Huntingtin (HTT) is a large (3,144aa) ubiquitously expressed, multi-domain protein with a molecular weight of 348 kDa, encoded by the *HTT* gene, located on chromosome 4p16.3 in humans (The Huntington’s Disease Collaborative Research Group, 1993). It exerts numerous biological roles, including a critical role in nervous system development, the ability to influence the production and transport of essential neurotrophic factors such as BDNF and its role in inter-neuronal communication and adhesion (Bates et al., 2015). *HTT* contains a rather genetically unstable CAG repeat region in the N-terminal (NT) region of the gene, which is considered “normal” when the repeat is between 6 and 35, but pathological in expansions ≥40 units: these encode a polyglutamine sequence (poly-Q tract), which exerts a critical role in the molecular pathogenesis of HD (Zuccato and Cattaneo, 2014; Bates et al., 2015; Bäuerlein et al., 2017; Tao et al., 2019). Structurally, HTT is characterized by clusters of helical α repeats, called HEAT domains, known to fulfil the role of molecular scaffolds. These HEAT domains are separated by unstructured regions that are subject to post-translational modifications which involve, in addition to phosphorylation and glycosylation, the genesis of protein fragments (as depicted in Figure 2B) (Zuccato and Cattaneo, 2014; Bates et al., 2015; Gelman et al., 2015; Tao et al., 2019). Among these, the NT fragment, which encompasses about 100 amino acids translated from the exon 1 of *HTT* gene, has been shown to play a key role in the pathogenesis of HD. *HTT* exon 1 encodes a 17 amino acid N-terminal segment, known as HTTNT or N17, comprising a poly-Q segment encoded by CAG repeats of varying length, and a proline rich domain (PRD) of 51 amino acids. The HTTNT stretch can be found in an “intrinsically disordered” conformation in the monomeric state, but it is also capable of acquiring a α-helical conformation upon self-association or after binding to membranes; in this context, the poly-Q sequence in the monomeric peptides tends to favour a condensed and disordered state; finally, the PRD within HTTNT is likely to exist in fluctuating segments of disordered helices and type II polyproline, a spatial conformation known to be a good binding motif and to foster highly dynamic protein-protein interactions (Bates et al., 2015; Gelman et al., 2015). Finally, it is worth mentioning that a considerable amount of experimental evidence has shown that HTT proteolysis and fragmentation represent a major factor in dictating the pathogenesis of HD. As such, those processes appear to be influenced by the expanded poly-Q segment. It is known, for instance, that with increasing length and concentration of the poly-Q repeat, the time required for nucleation of amyloid fibrils decreases (Scherzinger et al., 1999; Bates et al., 2015; Gelman et al., 2015; Bäuerlein et al., 2017; Pandey et al., 2018).

The toxicity induced by the NT fragment with the expanded poly-Q tract suggests a “gain of function” mechanism, where the nuclear translocation of these fragments with the formation of intra-nuclear inclusion bodies is observed, along with their cytoplasmic accumulation, which progressively leads to several cellular dysfunctions (Gunawardena et al., 2003; Gauthier et al., 2004; Lee et al., 2004; Pal et al., 2006; Suopanki et al., 2006; Ross and Tabrizi, 2011). To corroborate the hypotheses about the toxicity of the poly-Q tract, it was experimentally observed that the expansion of the latter is strongly related to the pathogenesis of eight other neurodegenerative disorders, including various spinocerebellar ataxias (Orr, 2012).

The turnover of HTT mainly depends on proteasomal degradation, but monomeric and polymeric forms can also be degraded by autophagy (Kegel et al., 2000; Ochaba et al., 2014; Martin et al., 2015; Rui et al., 2015; Menzies et al., 2017). Regarding proteasome-dependent degradation, biochemical analyses show that ubiquitin is present in the mutant HTT (mHTT) inclusion bodies (Hazeki et al., 2002; Ochaba et al., 2014; Rui et al., 2015) and mutant HTT itself can undergo ubiquitination (Waelter et al., 2001; Martin et al., 2015; Menzies et al., 2017). Furthermore, a number of genetic and/or pharmacological perturbations leading to the inhibition of the ubiquitin-proteasome system (UPS) can impact on the turn-over of HTT, ultimately leading to the accumulation of ubiquitinated mutant HTT and an increased presence within aggregates and inclusion bodies (Waelter et al., 2001; Zhou et al., 2003). In this context, when the proteasome-dependent system becomes saturated, the activation of autophagic pathways is more evident. Finally, in various HD models, it has been shown that the poly-Q tract activates autophagic pathways through the inhibition of the mTORC1 kinase complex: such an upregulation protects the cell from toxicity induced by the fragments themselves (Kegel et al., 2000; Martin et al., 2015; Menzies et al., 2017).

Nevertheless, the prolonged expression of aggregation-prone proteins including mutant HTT is detrimental and ultimately lead to the collapse of the proteostatic network: the levels of basal chaperones decrease with the progression of the disease, stress of the endoplasmic reticulum (ER stress) and activation of the unfolded protein response (UPR) becomes evident and both the ubiquitin-proteasome system (UPS) and autophagy are progressively compromised (Bates et al., 2015; Remondelli and Renna, 2017). Furthermore, recent reports suggest that the aforementioned protein aggregates can spread between neurons as well as between neuronal and glial cells (Cicchetti et al., 2014).

Although HD has been well established as a cell autonomous genetic disorder, recent experimental work, performed by using both *in vitro* and *in vivo* models, has shown that mutant HTT can be transmitted to neighbouring cells (Pecho-Vrieseling et al., 2014; Babcock and Ganetzky, 2015; Pearce et al., 2015; Jansen et al., 2017; Jucker and Walker, 2018; Pearce and Kopito, 2018), demonstrating potential prion-like infection and propagation properties (Cicchetti et al., 2014; Jeon et al., 2016). Interestingly, transcellular transmission of mutant HTT can also trigger the heterogeneous aggregation with wildtype forms of the protein (Babcock and Ganetzky, 2015; Pearce et al., 2015). Considerable experimental (Chen et al., 2002; Bhattacharyya et al., 2005; Kar et al., 2011) and computational efforts (Chen et al., 2016; Chen and Wolynes, 2017; Lakhani et al., 2010) have been directed towards identifying the molecular mechanism governing the homogeneous aggregation of HTT in a poly-Q dependent manner. In this context, the large majority of experimental evidence regarding poly-Q-dependent aggregation has been obtained from studies conducted on isolated poly-Q monomers and oligomers. However, the structural properties of monomeric poly-Q are still rather unclear and inconsistent (Długosz and Trylska, 2011). Indeed, experiments on single poly-Q tracts of different lengths and/or modified for solubility, suggest high conformational flexibility (a random coil, a collapsed structure, an *α*-helix, a *β*-sheet, or even a PP II helix) and a dependence of its structure on the local environment. It is generally believed that poly-Q aggregates adopt structures rich in *β*-sheet content, with longer poly-Q peptides being able to aggregate more rapidly (Długosz and Trylska, 2011). However, the heterogeneous aggregation of HTT species bearing different lengths of the poly-Q tract has been much less studied (Busch et al., 2003; Isas et al., 2017), despite its fundamental relevance for a possible prion-like disease propagation.

Protein oligomerization and aggregation within cells is a considerably more intricate process than the one which occurs and has been observed in isolated poly-Q tracts and can also be influenced by the intracellular environment. Hence, to better mimic the physiological conditions, some studies have used a number of other soluble proteins fused to poly-Q (Bulone et al., 2006), allowing one to infer the relative contributions of the poly-Q stretch and of the rest of the structure in modulating aggregation. For instance, molecular dynamics simulation studies have defined a positive correlation between the length of the poly-Q expansion and its probability to form a *β*-sheet enriched misfolded state (Lakhani et al., 2010). On the basis of these simulations, it was suggested that the surrounding regions might influence the formation of *β*-sheet structures within the poly-Q region (Lakhani et al., 2010). More recent simulations were able to define the poly-Q length-dependent, free-energy landscape of the homogeneous HTT Exon 1 oligomerization process (Chen et al., 2016; Chen and Wolynes, 2017). In particular, thermodynamic modifications of the structure determined by the addition of specific residues, which were capable of either enhancing or inhibiting oligomerization, were investigated (Chen and Wolynes, 2017). However, such studies have been limited to analysis of the poly-Q region and therefore the contribution made by post-translational modification to other regions is still largely not known.

### 1.3 Small HSPs: structure and function of HSPB1/HSP27

Small heat shock proteins (sHSPs or HSPBs) represent a highly conserved family of ATP-independent molecular chaperones that are able to recognise unfolded or misfolded proteins and to hold them in a folding-competent state until the ATP-dependent HSPs complete the refolding process (Haslbeck and Vierling, 2015). When correct folding is not achieved, sHSPs assist in the removal of these pro-aggregating intermediates by directing them towards degradation systems. In humans, the HSPBs family includes 11 members (HSPB1-11), with molecular mass ranging between 12 and 40kDa, capable of assembling into large oligomers of 4–40 subunits and to stably interact with non-native client proteins (Mchaourab et al., 2009; Arrigo, 2017; Muranova et al., 2019; Mymrikov et al., 2020; Vendredy et al., 2020). The N-terminal domain varies in length and sequence, while a stretch of residues located at the C-terminal (the C-terminal tail) has a conserved motif (Caspers et al., 1995; Boelens, 2020). Importantly, all the HSPBs family members also possess a conserved domain within the central region of their sequences, called the α-crystallin domain, which is critical for their chaperone activity (Caspers et al., 1995; Boelens, 2020).

HSPB1 (Hsp27) is a ubiquitously expressed small HSP, encoded by the *HSPB1* gene located on chromosome 7 in humans (Stock et al., 2003; Arrigo, 2017; Arrigo, 2018). From the structural point of view, HSPB1 includes a partially conserved N-terminal sequence, the α-crystallin domain in the central region and a flexible C-terminal domain (as schematically depicted in Figure 2B). HSPB1 is in dynamic equilibrium between high molecular weight oligomers and much lower molecular weight multimers. In a non-phosphorylated state, HSPB1 generates high molecular weight aggregates, ranging from 12 to 35 subunits (Haslbeck et al., 2005; Arrigo, 2017; Arrigo, 2018). Conversely, the phosphorylation of HSPB1 to serine residue 15, 78, and 82, by the kinase MAPKAP-2 activated by p38 (Kamradt et al., 2001), or by the use of the triple phospho-mimetic Ser-to-Asp, results in an important shift of the equilibrium towards the genesis of multimers (van Montfort et al., 2001) and in a functional alteration of the protein (Richter et al., 2010).

## 2 Centrifugation: theory and techniques

Centrifugation is a fundamental technique in biomedical sciences, as it allows one, by means of centrifugal force-based separation, to isolate and purify mixtures of biological particles immersed in a liquid medium. It is a key method for the isolation and analysis of cells, subcellular and extracellular components and fractions, as well as supra-molecular complexes and macromolecules, such as proteins, lipids or nucleic acids (Graham, 1997; Hinton and Mullock, 1997).

From the theoretical standpoint, the sedimentation rate of any particle (v) depends on the applied centrifugal field *g* (cm⁄s ^ 2), which in turn is determined by the radial distance, (r) of the particle from the axis of rotation (cm), multiplied by the square of the angular velocity, ω, of the rotor (rad⁄s):

$$G=\omega \hat{\;}\;2\;r$$

The centrifugal field is generally expressed in multiples of the gravitational field g (981 cm⁄s ^ 2) by means of the relative centrifugal field (RCF) units:

RCF=G / g

In a mixed liquid solution of biological particles, the sedimentation rate will depend not only on the centrifugal field applied, but also on the nature of the analysed particle, that is, on its density and size, as well as on the viscosity of the medium in which it is immersed. The sedimentation rate of a biological particle can also be expressed as a function of its sedimentation coefficient (s), where:

$$s=v\;/\;\left(\omega \hat{\;}\;2\;r\right)$$

Historically, the sedimentation coefficients of biological components have usually been expressed as Svedberg units (S): a Svedberg unit equals 10–13 s (Wilson and Walker, 1981). Typically, centrifugation can be conducted for either preparatory or analytical purposes. Preparative centrifugation allows the separation of cellular components such as vesicles, nuclear fractions, mitochondria, chloroplasts, proteins, etc. This preparative centrifugation can, in turn, be differential and in density gradient. Differential centrifugation is based on the sedimentation rate of biological particles as a function of their different size and/or density properties: in particular, higher density particles will settle more rapidly. In practice, the sample is evenly distributed in the tube to be centrifuged: during centrifugation, the various particles will move along the tube with their respective sedimentation speed. Following this procedure, the particles are separated by various centrifugation steps, increasing the centrifugal field applied each time, so as to obtain a pellet and a supernatant in each step. Specifically, the resulting supernatant fractions are subjected to an additional centrifugation at a higher speed and for a longer time, in order to separate the particles that have not been sedimented in the previous step. This process continues with further centrifugation steps until the biological sample of interest is isolated.

Differential ultracentrifugation has been used to carry out the experiments described in the method reported here; to date it remains the gold standard for the isolation of extracellular vesicles, including exosomes (Bonavita et al., 2023; D’Agostino et al., 2019; Doyle and Wang, 2019). A typical protocol for the isolation of exosomes (Bonavita et al., 2023) by differential ultracentrifugation requires: 1) a first 500 g centrifugation, which allows one to generate a pellet containing cellular debris and larger particles from the matrix; 2) filtration at 0.22 µm, followed by a second centrifugation at 100,000×g to further purify the matrix, allowing the removal of larger apoptotic bodies and extracellular vesicles; 3) finally, the smaller extracellular vesicles, including exosomes, are pelleted by ultra-centrifugation at 100,000×g. The yield of exosomes can be increased by extending the 100,000×g ultra-centrifugation times; however, it has been shown that if more than 4 h of ultra-centrifugation are used, significant mechanical damage to exosomes and other extracellular vesicles (EVs) can occur and, therefore, higher levels of contamination of soluble proteins in the final preparation can be observed (Vlassov et al., 2015).

In preparations where it is necessary to separate biological particles of similar size, but bearing different density, the preferred approach is to use the density gradient ultracentrifugation. The sample is first layered on the surface of a density gradient and, subsequently, a centrifugal force is applied: the sample particles pass through the gradient, which increases in density from top to bottom, with a certain sedimentation rate.

Density gradient centrifugation is defined as zonal when centrifugation is performed for a time sufficient for the particles to settle in discrete areas of the gradient, but not sufficient for sedimentation to the bottom of the tube. In this way it is possible to separate particles with similar density, but with different shapes and sizes. Centrifugation is carried out for a time that allows the biological particle to localize in a zone of the gradient with a density equal to that of the particle itself. Depending on the purpose with which this method is pursued, a large variety of gradients are available. Cesium chloride, for example, has been widely used for the separation of DNA bands and for the isolation of plasmids, nuclear proteins and viruses. While, for the separation of membrane vesicles deriving from tissue homogenates or cell cultures, ultra-pure sucrose free of DNase, RNase and protease is used (Graham, 1997; Hinton and Mullock, 1997).

Historically, a number of different cellular fractionation protocols followed by isopycnic ultracentrifugation have been established in order to address different needs and look at different cellular processes. The strictly controlled mechanical homogenisation in the absence of detergent is aimed at minimizing any alteration of the membrane composition and permeability as well as of the protein complexes, which would in turn impact on their relative density and on their partitioning properties. Equally a number of options are available in terms of gradient preparation, such as Percoll, Nycodenz, OPTI-PREP, metrizamide, once again depending on the cellular compartments (ER, Golgi, mitochondria, endosomes, autophagosomes, lysosomes or extracellular vesicles) and/or the protein complexes that one needs to enrich or isolate (Amodio et al., 2009; Koga et al., 2010; Greening et al., 2015).

In the laboratory we have worked on the development of a system to evaluate the oligomerization of several disease-relevant proteins by analysing the sedimentation profile obtained by means of a discontinuous sucrose gradient ultracentrifugation. In particular, we have opted for the discontinuous sucrose gradient for the analysis of mutant huntingtin since we have previously deployed the same approach to look at and confirm the phosphorylation-dependent change of oligomerization of HSPB1 (Lambert et al., 1999; Kostenko and Moens, 2009; Bolhuis and Richter-Landsberg, 2010; Arrigo, 2017), showing how the HSPB1 3D phospho-mimetic mutant is distributed in small/lighter oligomeric complexes, compared to the wild type (WT) and the 3A-HSPB1 mutant (Bonavita et al., 2023).

**FIGURE 1:**
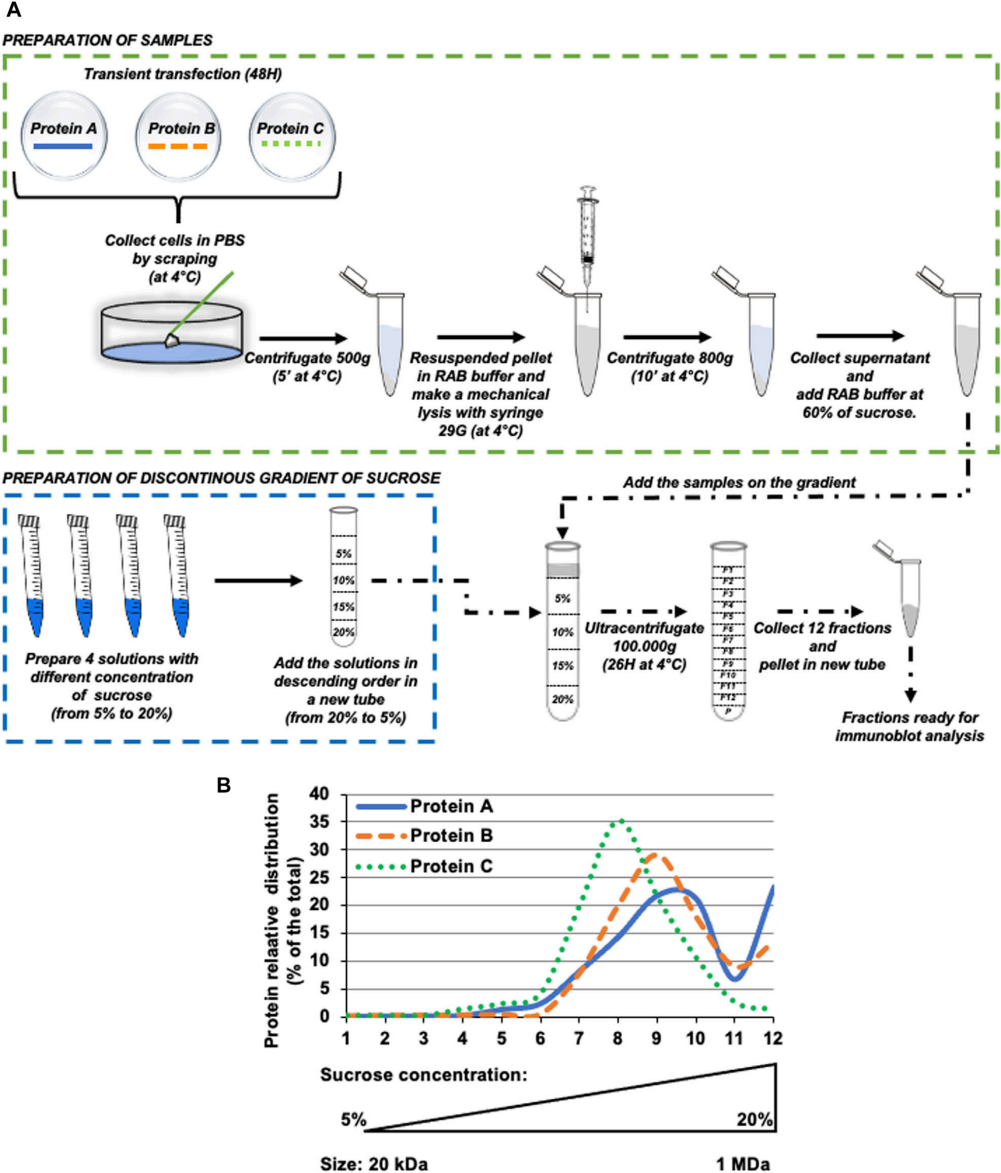
Schematic overview of basic principles and methodological analysis of protein oligomerization by isopycnic ultracentrifugation. **(A)** Schematic diagram of the complete experimental pipeline. Briefly, HeLa cells were transfected with protein of interest, then after 48 h cells were collected and processed as described in the Figure. The resuspended pellet was ultracentrifuged for 26 h at 100.000*xg* on a discontinuous sucrose gradient (ranging from 5% to 20% sucrose, weight/volume). After ultracentrifugation, 12 fractions were collected from the top of the tube and samples were analyzed by western blot. **(B)** Hypothetical protein (indicated as Protein (A-C) distribution profile upon western blot analysis of intracellular fractions obtained by isopycnic ultracentrifugation.

With this method, we demonstrate that it is possible to separate the protein oligomers on the basis of their density, which directly depends on the oligomerization state of the proteins, ranging from 20 kDa to 1 MDa (as depicted in Figure 1B, bottom of the representative graph). This methods article describes a novel protocol for analysing the oligomerization profile of huntingtin in mammalian cells. The method we have developed represents a sensitive and flexible tool to monitor HTT oligomerization and aggregation, which are autophagy-dependent and is suitable for siRNA and cDNA over-expression approaches, as well as for studies aimed at assessing the effect of small molecules and bioactive compounds. Furthermore, the isolated HTT-containing fractions may be also subjected to a number of downstream orthogonal applications, such as 2D/MS for proteomics research and interaction studies, as well as disease profiling, gene expression and signal transduction studies.

## 3 Materials and equipment

### 3.1 Material

• Dulbecco’s Modified Eagle’s Medium (DMEM) (D6546, Sigma Aldrich).
• Fetal bovine serum (FBS) (F0679, Sigma Aldrich).
• L-Glutamine (G7513, Sigma Aldrich).
• Penicillin/Streptomycin solution (P7539, Sigma Aldrich).
• Trypsin/EDTA solution (T4299, Sigma Aldrich).
• 75 cm^2^ Tissue culture flasks (Falcon cat #353136).
• 6-well plates (Corning, cat #3516).
• Cell scrapers.
• X-tremeGENE™ 9 DNA Transfection Reagent (Roche, Milan, Italy), according to the manufacturer’s instructions.
• Ready to use 1× PBS tablets (MicroGem, TL1006-500 ML).
• MLS-50 Swinging-Bucket Rotor (Beckman Coulter, cat. no. 367280) mounted on the Optima MAX-130K Ultracentrifuge (Beckman Coulter).
• Beckman Ultracentrifugation 5 mL polypropylene tubes (cat. No. 326819).
• Reassembly Buffer (RAB): 0.1 M Tris, 0.5 mM MgSO_4_, 1 mM EGTA, 2 mM DTT, supplemented with two tablets of protease inhibitors (Roche Complete™ Protease Inhibitor Cocktail, 54937500).
• For mechanical lysis: Insulin Syringe with Needle, 29G 1cc 1/2-Inch.
• Ready to use Lysis Buffer: 150 mM NaCl, 10 mM Tris–HCl pH 6.8, 1 mM EDTA pH: 8.0%, 10% glycerol and 1% Triton X-100, and supplemented with a tablet of protease inhibitor cocktail (Roche Complete™ Protease Inhibitor Cocktail, 54937500).
• Bench-top refrigerated microcentrifuge (Beckman).
• 1.5 mL Eppendorf tubes.
• 15 mL conical tubes.
• Vortex mixer.
• 3X Laemmli sample buffer: 188 mM TRIS-HCl pH: 6.8, 3% SDS, 30% glycerol, 0.01% w/v Bromophenol Blue, 15% Beta-Mercaptoethanol (Bio-Rad).
• Protein concentration evaluation: Quick Start™ Bradford Kit 2 (cat. No. 500-0205).
• Mini-protean gel tanks and casting units (Bio-Rad).
• Power pack (Bio-Rad).
• Trans blot SD semidry transfer cell (Bio-Rad).
• PageRuler™ Plus Pre-stained Protein Ladder, 10–250 kDa (Thermo-Scientific, cat. No. #26619).
• PVDF membrane (Immobilon-P; Millipore, cat. No. #IPVH00010);
• Extra-thick filter paper (Bio-Rad, cat #1703965).
• Methanol (Sigma Aldrich, cat #34860).
• Anti-FLAG antibody (M2, mouse monoclonal; Sigma-Aldrich (cat. No. F1804).
• Mouse monoclonal anti-huntingtin antibody (Millipore, United States cat. #MAB2166).
• Peroxidase conjugated anti-mouse antibody (Sigma-Aldrich, cat. No. A4416).
• Clarity Western ECL Substrate was from Bio-Rad (California, United States; #170-506).
• High-performance chemiluminescence film (Hyperfilm ECL, GE Healthcare). (Catalogue number: 28-9068-36)
• Hyper-cassette (GE Healthcare).
• RP X-OMAT Film Processor, Model M6B (Developer, Kodak).
• ImageJ Software.

### 3.2 Solutions

• RAB Buffer: 0.1 M Tris, 0.5 mM MgSO_4_, 1 mM EGTA, 2 mM DTT, pH: 7.5; supplemented just before use with two tablets of protease inhibitors (Roche Complete™ Protease Inhibitor Cocktail, 54937500).
• 10X B-buffer and 1X B-Buffer 1% Triton X-100: 150 mM NaCl, 10-mM Tris-HCl pH: 7.4, 1 mM EDTA pH 8.0 containing 0.6% CHAPS in the presence of protease inhibitor cocktail
• 2X Lysis Buffer: 20 mM Tris-HCl, pH: 6.8, 137 mM NaCl, 1 mM EGTA, 1% Triton X-100, 10% glycerol.
• The 2X buffer is stored at 4°C. 1X lysis buffer is prepared freshly with the addition of sterile distilled water and supplemented with protease inhibitor cocktail (Roche) just before use.
• SDS-PAGE Resolving gel (12%) To make 20 mL: 8 mL 30% (37.5:1) acrylamide:bis-acrylamide solution, 5 mL 1.5 M Tris-HCl, pH: 8.8, 200 μL 10% sodium dodecyl sulphate (SDS), 200 μL 10% ammonium persulphate (APS), 8 μL N,N,N,N_0_- tetramethylethylenediamine (TEMED) and 6.6 mL distilled water.
• SDS-PAGE Stacking gel (5%) To make 5 mL: 3.4 mL 30% (37.5:1) acrylamide:bis-acrylamide. solution, 0.63 mL 1 M Tris- HCl, pH: 6.8, 50 μL 10% SDS, 50 μL 10% APS, 5 μL TEMED, and 3.4 mL distilled water.
• Water-saturated isobutanol: Combine equal volumes of distilled water and isobutanol in a glass bottle, mix on a stirrer overnight, allow to separate, and store at room temperature. Use the top (butanol) layer.
• 3X sample loading buffer (Laemmli buffer): 187.5 mM Tris-HCl, pH 6.8, 6% w/v SDS, 30% glycerol, 15% β-mercaptoethanol, 0.03% w/v bromophenol blue.
• 10X running buffer and 10X transfer buffer: 250 mM Tris-HCl, 1.92 M glycine. For 1X running buffer dilute 100 mL of 10X buffer with 890 mL of distilled water and 10 mL of 10% SDS. For 1X transfer buffer dilute 100 mL of 10X buffer with 200 mL of methanol and 700 mL of distilled water.
• Blocking buffer: 5% (w/v) non-fat dry milk, 0.1% (v/v) Tween-20 in 1X PBS.
• 60% (w/v) Sucrose solution: Add RAB buffer to 60.0 g of sucrose to a final volume of 100 mL, final pH: 7.5.
• Heat gently and stir continuously until sucrose is fully dissolved.

*Note: It is important to note that to avoid any possible alteration in either the concentration or in the ionic strength of the homogenization (RAB) buffer, the 60% sucrose solution (w/v) must be prepared by dissolving sucrose in RAB Buffer. Hence, the pH of the buffers used in our protocol are entirely compatible with the expected range of cytosolic pH of eukaryotic cells (pH: 7.6–7.8).

## 4 Methods

### 4.1 Cell culture

All cell lines used in the protocols described here can be cultured under standard conditions.

1. HeLa cells are cultured in DMEM supplemented with 10% FBS, 100 U/mL penicillin/streptomycin, and 2 mM L-glutamine at 37°C, 5% CO_2_ with humidity.
2. Cells are kept in standard growing conditions until they reach 80%–90% confluency
3. To passage the cells, the medium is removed from the flask and cells are rinsed once with sterile 1x PBS.
4. 2.5 mL of 1X trypsin-EDTA solution is added to the cells and incubate at 37°C for 5 min, or until the cells are detached from the bottom of the flask.
5. The trypsin activity is quenched by adding 8 mL of fresh complete culturing medium.
6. Cells are then centrifuged at 500 *g* for 5 min at room temperature and cellular pellet resuspended in an appropriate volume of complete culturing medium.
7. The cell resuspension is transferred to the desired/required dish/flask, depending on your experiment.

Note: Depending on the cell type and duplication time, most of the cell lines can be grown and routinely split in fresh supplemented medium and every 3–5 days in 1:5 or 1:10 ratio and maintained at 37°C, in a 5% or 10% CO_2_ incubator with humidity.

#### 4.1.1 Preparation of Solutions

1x Phosphate-Buffered Saline (PBS): dissolve the adequate number of 1x PBS tablets (P4417, Sigma-Aldrich) in ultrapure sterile water.

The Sucrose Cell Separation buffer is prepared as a filtered 60% solution in RAB Buffer. Prepare 4 gradient solutions using the RAB buffer as a dilution buffer, as indicated in Table 1.

**TABLE 1:** Discontinuous sucrose gradient preparation. For each layer (from 1 to 4), it is reported the required volumes of 60% sucrose cell separation medium and RAB buffer to be combined in order to obtain the 5%–20% gradient solutions.

Fractions	Sucrose gradient cell separation medium volume (μL)	Gradient dilution RAB buffer volume (μL)	Final volume (μL)	Final gradient (%)
Layer 1	100	1100	1200	5%
Layer 2	200	1000	1200	10%
Layer 3	300	900	1200	15%
Layer 4	400	800	1200	20%

* Note: each volume (1200 μL) is needed (in excess) for and refers to the volume required for one sample preparation (1000 μL). It is therefore recommended to prepare master mix stocks of each solution (namely, fractions 1–4 ranging from 5% to 20% sucrose, respectively, as reported in Table 1).

#### 4.1.2 Experimental cell culture setup and preparation of detergent-free cell homogenate

The detailed protocol described below can be applied to a variety of standard cell lines, such as HeLa, HEK293T, SK-N-SH, mouse embryonic fibroblast (MEF) cells, as well as primary cultures, such as human macrophages and mouse cortical neurons. Equally, we provide, in the result section, supporting evidence that it can also be implemented for the analysis of conditioned medium and EVs-derived from these cells.

**FIGURE 2:**
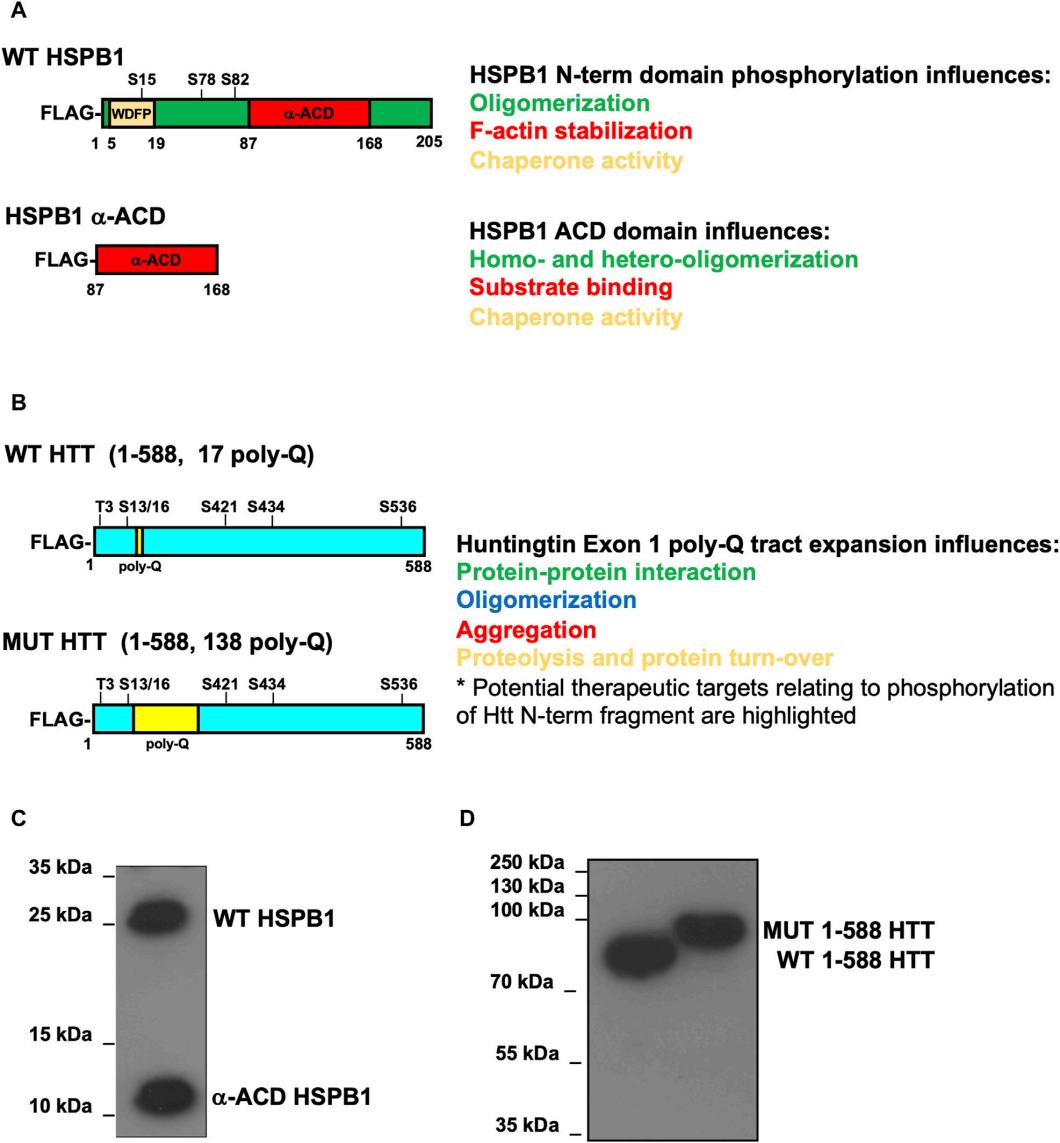
Schematic overview of the expression constructs used in this method article. **(A,B)** Schematic representation of transfected proteins WT-HSPB1, HSPB1 ACD domain, full-length HTT, WT-HTT and MUT-HTT. **(C,D)** Western blot analysis using anti-FLAG antibody to detect WT-HSPB1, HSPB1 ACD domain, WT-HTT and MUT-HTT expression in HeLa cells. The different MW between WT and mutant HTT is mainly explained because of the presence in the mutant protein of the expanded polyglutamine tract (namely, 138 poly-Q), compared to the wild-type protein, bearing the 17 poly-Q tract. In addition, the presence of the expanded poly-Q is generally responsible for the acquisition of a number of post-translational modifications (such as ubiquitylation), which can further impact on the molecular weight of the protein.

NOTE: if the supernatant is cloudy, once it has been transferred to other tubes, a further round of centrifugation is performed (800*xg* for 10 min at 4°C).

1. HeLa cells are cultured in DMEM, supplemented with 10% fetal bovine serum (FBS), 100 U/mL penicillin/streptomycin and 2-mm L-glutamine at 37°C in a humidified atmosphere of 5% CO_2_ and 95% O_2_.
2. Transient transfection can be performed with a number of standard and commercially available transfection reagents. Among the others, the X-tremeGENE™ 9 DNA Transfection Reagent (Roche, Milan, Italy), used according to the manufacturer’s instructions, resulted in an extremely high and reproducible over-expression of our proteins of interest. This was particular important, in consideration of the large amount of material required for this kind of biochemical analysis.
3. HeLa cells are transfected with FLAG-tagged plasmids encoding for WT-HSPB1, HSPB1 ACD domain, WT 1-588/HTT and MUT 1-588/HTT, as schematically depicted in Figures 2A,B.
4. After 48 h of transfection in 100 mm plates of HeLa cells, aspirate the culture medium on ice.
5. Wash the dishes twice with 10 mL of pre-chilled 1x PBS.
6. Detach cells manually with the aid of a cell scraper, resuspend them in 1 mL of 1x PBS, and transfer into 1.5 mL Eppendorf tubes placed on ice.
7. Centrifuge the obtained samples at 500*xg* for 5 min at 4°C.
8. Following centrifugation, discard the supernatants, and flick the pellet gently to aid the resuspension of the cell pellet.
9. Resuspend the pellets in 800 µL of RAB Reassembly Buffer.
10. Mechanical lysis is obtained by repeated uptake and ejection using 29-gauge 1 mL syringes, in the cold room. Two repeats of 10 rounds of syringing are performed, interspersed with a short rest of the sample on ice.
11. To remove the membranes and isolate the supernatant corresponding to the cell lysate, the samples are centrifuged for 10 min at 800xg at 4°C.

12. Aliquots from clarified supernatants are stored as INPUT samples for further analysis.

#### 4.1.3 Preparation of homogenates for discontinuous sucrose density gradient ultracentrifugation

The concentrations of the sucrose gradient solutions required for the fraction isolation may require optimisation, depending on the cell type, the size or the nature of the multi-protein and/or macromolecular complex one might be interested in analysing.

For the procedure described below, it is possible to use for enrichment either an open- or closed-top ultracentrifuge tubes (volume: 5–10 mL). The ultracentrifuge must be equipped with an adequate swinging rotor.

1. In an ultracentrifuge tube, prepare a discontinuous density gradient by carefully overlaying the prepared gradient solutions (see Section A and Table 1), proceeding in descending concentrations (i.e., from the bottom to the top layer). In this specific case, first add the 20% gradient solution and then the 15%, 10%, and 5% gradient solutions, respectively.
2. Mix the prepared cell or extracellular media homogenate (Section B) with the 60% sucrose solution to make a final concentration of 5% sucrose cell homogenate. For example, add 1100 μL of homogenate to 100 μL of 60% sucrose solution.
3. Overlay the sample containing the 5% sucrose cell homogenate on top of the density gradients prepared in Step 1 (as detailed in Figure 1).
4. Ultracentrifuge the samples at 100,000×*g* for 26 h at 4°C.

#### 4.1.4 Collection of fractions from sucrose density gradients

It is extremely important and therefore strongly recommended that all the steps described in this section are performed at 4°C, to preserve the quality of the collected samples.

After centrifugation, samples can be collected in the cold room from the top surface of the gradient (starting, therefore, from the 5% sucrose solution) with the aid of a pipette, as follows:

1. Starting from the top of the tube, remove each defined fraction from 1 to 12 (i.e. 450 μL/fraction, total volume: 5400 μL).
2. The sample can be transferred into a 1.5 mL Eppendorf tube and add 150 μL of 3X Laemmli buffer, vortex thoroughly and leave the tubes on ice for 30 min to allow the lysis.
3. Add 200 μL of complete Lysis buffer to resuspend the pellet and leave on ice for 30 min.
4. At this stage, samples are ready for SDS-PAGE or can be snap-frozen and kept at −80^o^C until downstream processing. However, we strongly recommend proceeding immediately with SDS-PAGE and western blot analysis, as freezing and thawing may compromise the integrity of the fractions.

### 4.2 Preparation of samples for western blot analysis

#### 4.2.1 SDS-PAGE

1. Prepare the gels for SDS-PAGE by first pouring the resolving gel solution (10%–13.5% acrylamide, depending on the protein of interest) between a gel plate with a 1.5-mm spacer and a glass plate sandwich held together in the gel casting unit. Normally, the resolving gel is poured up to 75% of the length of the plates, leaving space (25%) for the stacking gel.
2. Overlay approximately 500 μL of water-saturated butanol on the surface of the resolving gel, once the gel has polymerized this should be washed off with distilled water.
3. Pour the stacking gel over the solidified resolving gel matrix and insert a 10- or 15-well comb. After polymerization of the stacking gel, remove the combs and rinse the wells with ultrapure distilled water.
4. Set the gels into the SDS-PAGE apparatus and add 1X Tris-Glycine running buffer containing SDS.
5. In the first lane, load pre-stained molecular weight markers. Then load the protein samples into the wells.
6. Run the gels at a constant voltage (100–130 V) until the protein ladder marker bands migrate to the desired position.

### 4.3 Semidry transfer of proteins and western blotting

In this section, a detailed description of the procedure to execute the fast (45′-1 h) and highly efficient semidry protein transfer, however samples can also be subjected to standard wet transfer without any overt decrease in the yield or the overall quality of results obtained.

7. Cut PVDF membranes and activate them by soaking in 100% methanol for 45 s.
8. Equilibrate them in 1X transfer buffer along with extra-thick 3 MM filter papers.
9. Remove the gel from the glass plates and equilibrate in the transfer buffer.
10. Assemble the gel-transfer unit in the following order: one filter paper at the base of the transfer plate, then the PVDF membrane, followed by the gel and finally 2 more filter papers on the top.
11. For each of the sandwich layering step described above, roll the surface gently with a plastic pipette, to remove any air bubbles.
12. Place the upper plate and connecting the lid, transfer at a constant voltage 980–100 V) for approximately 1 h.
13. Disassemble the sandwich and remove the PVDF membranes from the transfer apparatus, rinse them with fresh 1x PBS and then incubate them in 20–30 mL of 5% milk 1x PBS (blocking solution) on a rocking platform for 30′–1 h at room temperature.
14. Discard the blocking buffer and add primary antibodies diluted in 10–20 mL of blocking buffer (anti-FLAG at 1:5000 and anti-huntingtin at 1:1000 dilution for detecting recombinant HTT, recombinant FL- or ACD-HSPB1 and endogenous huntingtin, respectively) and incubated overnight on a rocking platform in a cold room at 4°C.

We are entirely aware that under most of the standard experimental procedures (for instance upon over-expression or in case of extremely abundant proteins), western blot analysis can also be carried out by incubating the membranes with the primary antibody solutions for just 1–2 h at room temperature. Nevertheless, in view of a number of already mentioned considerations regarding the amount of material and the instability of these complexes, it is strongly recommended to incubate the membranes with primary antibodies overnight to obtain the highest and most reproducible results.

15. Wash the membranes three times with 1x PBS Tween-20 solution, for 10 min each on a rocking platform at room temperature.
16. Add the appropriate secondary antibody solutions (HRP-conjugated anti-mouse IgG, diluted 1:5000 in blocking buffer) and incubate for 1–2 h on a rocking platform at room temperature.
17. Wash the membranes three times with 1x PBS Tween-20 solution, for 10 min each on a rocking platform at room temperature
18. Perform ECL reaction: depending on the size of the membrane, combine 1–2 mL each of the detection reagents 1 and 2 of the ECL Western blotting detection kit (Bio-Rad) and add this to the immunoblots for 5 min, ensuring an appropriate coverage of the entire surface of the membrane.
19. Discard the detection solution and wrap the immunoblots in cling film then place them into a hypercassette.
20. In a darkroom expose the blots to Hyperfilm for the appropriate exposure times
21. Develop the films in an automated developer.

### 4.4 Densitometric analysis

The quantitative analysis of immunoblots can be performed with ImageJ/Fiji software. In the results presented herein, the total amount of signal has been set to 100 and the relative levels of proteins in the different fractions has been expressed as a percentage of the total. the statistical significance (*p* values) can be determined by Student’s t-test or, where appropriate, by Anova Factorial test using StatView 4.3 software (Abacus Concepts).

## 5 Results

### 5.1 Oligomerization profile of HSPB1 and ACD/HSPB1

**FIGURE 3:**
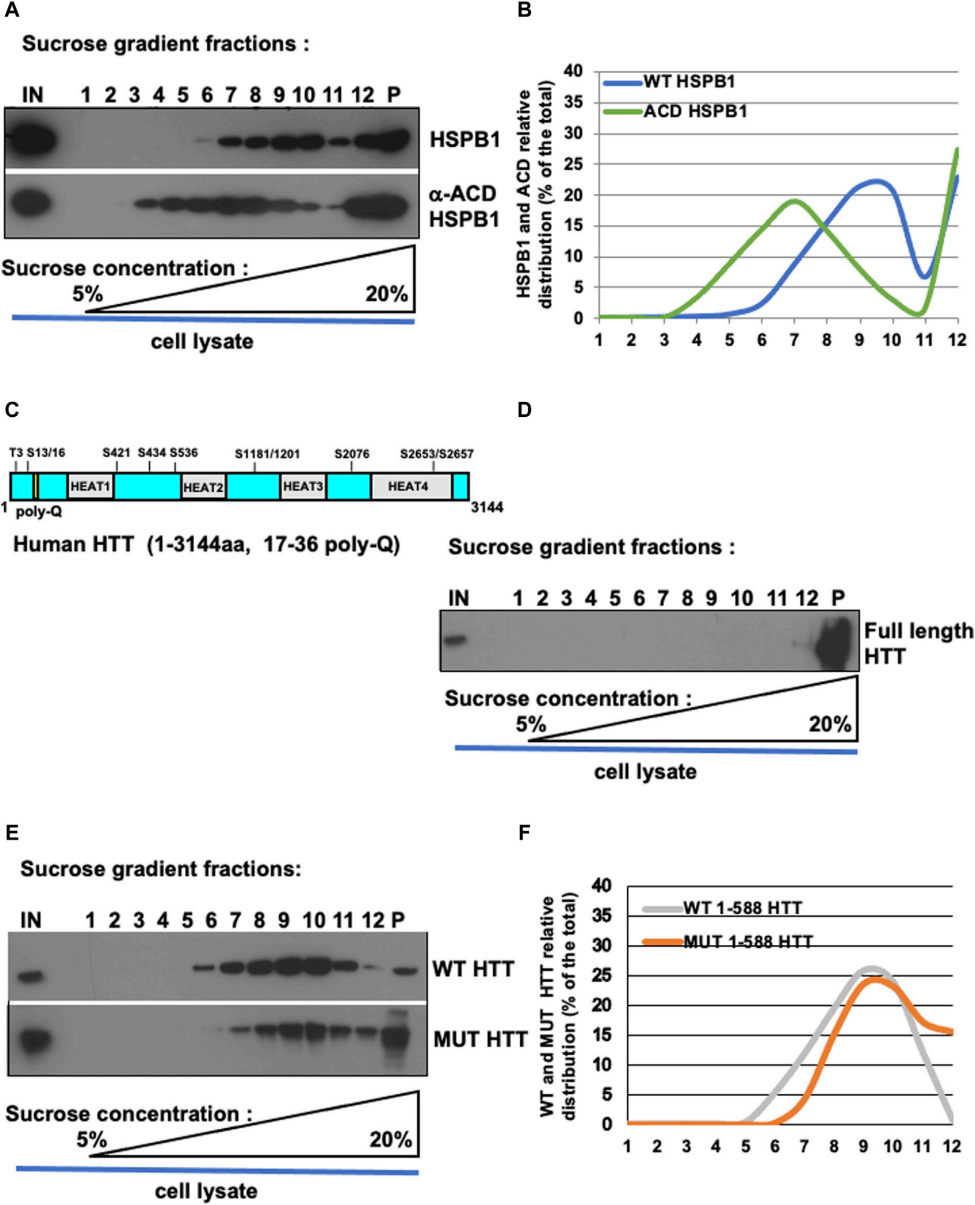
Oligomerization profile of HSPB1, ACD/HSPB1, full-length HTT, WT and MUT HTT by isopycnic ultracentrifugation. **(A)** Western blot analysis of sucrose gradient fractions of WT-HSPB1 and HSPB1 ACD domain. Post-nuclear supernatant fractions obtained from HeLa cells incubated were analysed by discontinuous sucrose gradient followed by SDS-PAGE and immunoblotting to detect the indicated proteins. **(B)** Representative analysis of HSPB1 and ACD relative distribution (% of the total) in 1–12 sucrose gradient fractions. The percentage of total protein contained in each fraction is reported on the *left scale*. the percentage (w/v) of sucrose is reported on the bottom side of the diagram. **(C,D)** Western blot analysis of sucrose gradient fractions of full-length HTT. **(D)** Full-length HTT relative distribution (% of the total) in sucrose gradient fractions. **(E)** Western-blot analysis of sucrose gradient fractions of WT-HTT and mut-HTT. **(F)** Representative analysis of WT-HTT and mut-HTT relative distribution (% of the total) in sucrose gradient fractions.

To validate our protocol, HeLa cells were transiently transfected with over-expression constructs encoding a FLAG-tagged version of either the full-length WT HSPB1 or the alpha-crystallin domain (ACD) of HSPB1 (Figure 2A). 48 h after transfection, cell homogenates were prepared as detailed in (namely, subsection 4.1.3 and schematically depicted in Figure 1A). Isopycnic ultracentrifugation (5%–20% sucrose gradient) of cell homogenates was performed to characterize the oligomerization state of the two different HSPB1 variants. Western blot analysis of the fractions collected from HeLa cells over-expressing the full length WT HSPB1, show how the protein was mainly present in its high molecular weight oligomeric form (fractions 7–12, with a peak in the region 9–10), whereas the ACD HSPB1 was represented in the cells also as smaller oligomers fractions 3–12, with a peak in the region 6–8, and a right hand-side shoulder), as expected (Figures 3A,B). Consistent with what previously reported (Bonavita et al., 2023), these results indicate that as such, this sucrose density ultracentrifugation-based fractionation method is remarkably sensitive and is therefore suitable to analyze and determine the oligomerization state of these oligomeric proteins.

### 5.2 Oligomerization profile of wild-type and mutant huntingtin

To test whether this protocol could be used also to study proteins able to form aggregates, such as huntingtin, homogenates were prepared from either parental HeLa cells (to look at endogenous HTT protein) or from HeLa cells transiently transfected with over-expression constructs encoding for the FLAG-tagged N-terminal fragment (1–588) of either wild type huntingtin (bearing a 17 poly-Q within the Exon 1) or the mutant, with a 138 poly-Q expansion (Figures 2B–D). Cell extract was loaded on sucrose gradient and after isopycnic ultracentrifugation the different fractions were analyzed by immunoblot. Interestingly, the full-length, endogenous HTT was found only in the pellet and in one high density fraction (namely, fraction 12 in Figures 3C,D), indicating as expected the propension of the protein to form and maintain very high molecular weight oligomerization state in cells. The WT HTT N-terminal fragment was present in high molecular weight fractions (5–11, showing a peak in fractions 9–10; strikingly, the distribution of mutant HTT was significantly shifted toward the high molecular weight fractions (namely, fractions 7–12), with in addition an increased presence in the pellet (indicated as P, Figures 3E,F). We also performed size exclusion chromatography (SEC) analysis on total lysates obtained from either wild-type or mutant huntingtin transiently-transfected cells (as reported in Supplementary Info). To this aim, cell extracts were prepared by mechanical homogenization in RAB buffer and run through a size-exclusion chromatography column. 30 fractions were collected and aliquots from each fraction were analysed by western blot. We were able to detect both WT and MUT HTT (Supplementary Figures S2B, S3B, respectively). Interestingly, the WT HTT was detectable between fractions 9 and 14, which range from 840 kDa to 100 kDa (Supplementary Figure S2B, blue dotted-line box), whereas the MUT HTT was detectable between fractions 8 and 14, which range from 905 kDa to 100 kDa (Supplementary Figure S3B, red dotted-line box). Along with the monomeric species, upon SEC analysis it was possible to detect for both the WT and MUT protein the presence of additional species, likely dimeric or even (homo- or hetero-) multimeric species. In addition, from a qualitative standpoint, it was also possible to observe a tendency of the MUT variant (bearing the expanded poly-Q tract), to generate protein complexes with a relatively higher molecular weight, compared to the WT protein. All these data are entirely consistent with the results derived from our ultracentrifugation-based analytical method, as well as with what has been previously reported in the literature (Bäuerlein et al., 2017; Chen and Wolynes, 2017; Pandey et al., 2018; Tao et al., 2019). Collectively, these results are in line with the expected higher oligomerization state displayed by the mutant HTT, compared to wild type protein.

### 5.3 Effect of full length HSPB1 and ACD/HSPB1 over-expression on mut HTT oligomerization by isopycnic ultracentrifugation

**FIGURE 4:**
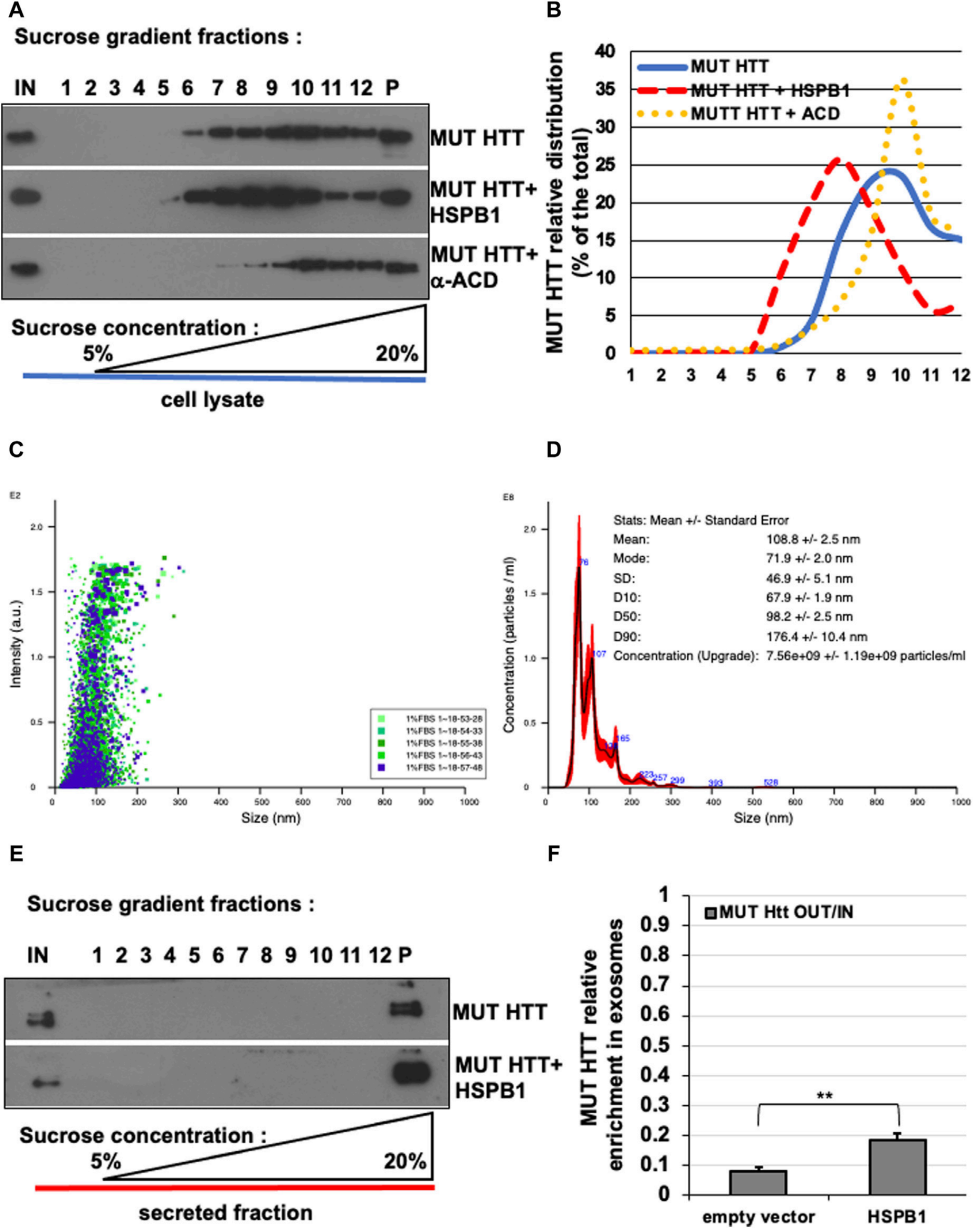
Effect of HSPB1 and ACD/HSPB1 over-expression on MUT-HTT oligomerization by isopycnic ultracentrifugation. **(A)** Western blot analysis of sucrose gradient fractions of MUT-HTT, mut-HTT + HSPB1, and MUT-HTT +α-ACD. **(B)** Representative analysis of mut-HTT relative distribution in the different conditions (% of the total) in sucrose gradient fractions. **(C,D)** Nanoparticle tracking analysis plots **(C)** and **(D)** report of the concentration and particles size average distribution of EVs derived from HeLa cells transiently transfected with MUT-HTT. **(E)** Western-blot analysis of sucrose gradient fractions of EVs of MUT-HTT and MUT-HTT + WT-HSPB1. **(F)** Representative analysis of EVs of MUT-HTT and MUT-HTT + WT-HSPB1 relative distribution (% of the total) in sucrose gradient fractions. The percentage of total protein contained in each fraction is reported on the *left scale*. the percentage (w/v) of sucrose is reported on the bottom side of the diagram. The histogram in F reports the MUT HTT relative enrichment in P100 fractions upon HSPB1 over-expression, compared empty vector transfected cells. Significance was assessed by factorial ANOVA. (*n* = 3; **p < 0.01).

A large number of studies have demonstrated how the ubiquitously expressed small heat shock protein HSPB1 is capable of reducing the aggregation propensity and inducing the clearance of several disease-associated mutant proteins such as HTT, α-synuclein and SOD1 (Wyttenbach et al., 2002; Nafar et al., 2015; Muranova et al., 2019; Chaplot et al., 2020; Bonavita et al., 2023). Our data confirmed and integrated such a scenario, showing how the over-expression of HSPB1 is capable of reducing the aggregation and promoting the EVs-dependent unconventional secretion of mutant HTT (Bonavita et al., 2023). For this reason, we transfected Hela cells with MUT HTT alone or in combination with WT HSPB1 or αACD HSPB1. The isopycnic ultracentrifugation-based analysis allowed us to appreciate the shift towards lower molecular weight of the MUT HTT in the presence of WT HSPB1, compared to MUT HTT alone. Interestingly, the over-expression of the αACD-HSPB1 induced a shift of the MUT HTT toward the high molecular weight oligomerization state (Figures 4A,B).

We also tested the usefulness of this method for the analysis of proteins extracted from isolated exosomes. Nanoparticle tracking analysis of the isolated EVs showed that the diameter of the particles ranged from 70 to 180 nm, with a mean value of 108.8 ± 2.5 nm, which is entirely compatible with the expected size range of exosomes and EVs (Figures 4C,D). We isolated exosomes from cells transfected with MUT HTT alone or in combination with WT HSPB1. Sucrose density gradient centrifugation of exosomes containing MUT HTT show that the protein is present only in the pellet (P), as one would expect in view of the small diameter and high density of EVs. In addition, we showed that the amount of mutant HTT contained in these structures was increased in EVs obtained from HeLa cells over-expressing WT HSPB1 (Figures 4E,F). These data are in line with our previous results describing the functional role exerted by HSPB1 in regulating the unconventional secretion of mutant HTT (Bonavita et al., 2023).

Collectively, these results provide solid proof of principle of the dynamic range of this isopycnic ultracentrifugation-based method and demonstrate how this can be used to analyse any functional variation in the oligomerization state of N-terminal fragment of mutant HTT.

### 5.4 Troubleshooting and additional information regarding time constraints

The experimental design and the timeline for the experiments described in this method article are very much dependent on the biological process under investigation. As an example, small molecules or drugs may only need to be added to cells for hours, whereas siRNA-based knockdown experiments may take several days to achieve a functional effect. Regardless, once the cells have been treated in the desired way for western blot analysis of MUT HTT, preparation of samples takes 2–4 h. The gels can then be run and transferred and put into primary antibody overnight on day 1. Western blot analysis can therefore be completed, ECL to be performed and the membranes exposed to the autoradiography film on day 2.

## 6 Discussion/commentary

HD is caused by a mutation resulting in the expansion of in the CAG triplet region of the Exon 1 of the *HTT* gene, which is characterised by an increased aggregation of HTT that in turn leads to progressive neurodegeneration and neuronal loss in the affected patients. The propensity toward aggregation is usually determined by the presence of an expanded poly-Q region with at least 36 repeats. Over the last 3 decades, the precise pathobiological role of these HTT-positive aggregates has been the subject of a long-standing debate in medical genetics. However, the progressive accumulation of these aggregates, which exert both loss- and gain-of-function effect, is now widely accepted as a prerequisite for neurotoxicity, as recently reviewed (Zhao et al., 2016). Hence, a deeper understanding of the multiple molecular mechanisms underlying HTT aggregation is considered a key instrumental factor to identify potential novel modulators capable of interfering with the process and alleviating the disease pathology (Chen and Wolynes, 2017). Notably, most of the scientific reports regarding this topic have been obtained from studies focusing on the homotypic aggregation of single species of mutant HTT bearing extended poly-Q tracts. Although less data are currently available regarding the heterotypic aggregation of mutant and wild type forms of the protein, a phenomenon that was firstly described by *in vitro* experimental approaches (Busch et al., 2003), this has been the focus of renewed attention since the ability of mutant HTT to undergo transcellular spreading was reported both in *D. melanogaster (* Babcock and Ganetzky, 2015; Pearce et al., 2015; Pearce and Kopito, 2018) and mouse brain models of the disease (Pecho-Vrieseling et al., 2014).

In this method article, we have developed and adapted a robust and standardised biochemical approach to follow by isopycnic ultracentrifugation the oligomerization profile of mutant HTT. Before moving to the analysis of mutant HTT, we have provided a proof of principle by looking at the oligomerization profile of the full length, wild type HSPB1, as well as of the ACD-HSPB1 fragment (Figures 3A,B). Consistent with this, we have previously deployed the same approach to look at and confirm the phosphorylation-dependent change of oligomerization that HSPB1 can be subjected to (Lambert et al., 1999; Kostenko and Moens, 2009; Bolhuis and Richter-Landsberg, 2010; Arrigo, 2017), showing how the HSPB1 3D phospho-mimetic mutant is distributed in small/lighter oligomeric complexes, compared to the wild type (WT) and 3A-HSPB1 mutant (Bonavita et al., 2023). It is also worth noting how in our experimental set up the large proportion of recombinant proteins are actually soluble and non-aggregated, as previously assessed by performing solubility assays (Bonavita et al., 2023). Hence, the protein complexes which are detected in the lighter (5%–10%) fractions of the gradients can be considered lower molecular weight oligomeric complexes. Among the other approaches available, size exclusion chromatography (SEC) is a well-established protocol, which has been used to assess the oligomerization of HSPB1 (Arrigo, 2018).

Interestingly, in a recent study regarding the mechanisms regulating the formation of HSPBs hetero-oligomeric species, by using size exclusion chromatography to look at the molecular configuration of HSPB1 in HeLa cells, the authors could observe how the protein size distribution ranges from 17 to 670 kDa, with the largest portion being present, as expected, from 150 to 670 kDa (Mymrikov et al., 2020). Consistent with this, our data show a significant difference in the oligomerization profile between the full length HSPB1 and the ACD-HSPB1 recombinant protein, which is still able to oligomerize and interact with client proteins, although displaying different partitioning properties. In this regard, the major challenge for applying SEC analysis downstream to our fractionation protocol would be the excessive dilution of the samples (i.e., the collected fractions), since the volumes required for the western blot analysis (namely, 30 μL out of 600 μL for each fraction; ratio 1:20, volume/volume) would result by far too diluted if prior subjected to SEC, likely compromising the possibility to detect (at least some of) the different oligomeric species, in particular the underrepresented ones. Nevertheless, such a method could definitely be applied on recombinant proteins for instance, which is something we might consider for future studies.

By means of this experimental strategy, we were able to assess the intracellular oligomeric status of the N-terminal fragment of both the wild type and mutant forms of HTT, which is critical for the HD pathobiology. In agreement with other scientific evidence, our data suggest how the pathological HTT aggregation might be generated by both heterogeneous and homogeneous oligomerization events of HTT proteins. Our experimental data confirm the evidence that the poly-Q residues represent a critical factor in contributing to the higher oligomerization of mutant HTT, which is key to the toxic aggregation of the protein and typically differ from those determining the conventional oligomerization of the wild type HTT protein.

A number of membrane-related functions have been reported for HTT, including signalling pathways, vesicular trafficking (Velier et al., 1998; Gunawardena et al., 2003; Gauthier et al., 2004; Lee et al., 2004; Pal et al., 2006) and autophagy (Ochaba et al., 2014; Gelman et al., 2015; Martin et al., 2015).

In addition to these physiological roles, membrane interaction is likely also of relevance in disease. Membrane interaction can potently accelerate aggregation and fibril formation of mutant HTT, in a misfolding process that can cause membrane damage and the formation of potentially toxic misfolded species (Kim et al., 2001; Pandey et al., 2018; Suopanki et al., 2006; Tao et al., 2019; Velier et al., 1998).

We have optimised this experimental approach to characterise the oligomerization properties of WT and MUT 1–588 N-terminal fragment of HTT. In a number of different cellular fractionation protocols followed by isopycnic ultracentrifugation, the strictly controlled mechanical homogenisation in the absence of detergent is aimed at minimizing any alteration of the membrane composition and permeability as well as of the protein complexes, which would in turn impact on their relative density and on their partitioning properties. Hence, it is worth noting that one of the potential limitations of our approach might be the inability to detect the membrane-associated portion of mutant HTT, for instance following to S-palmitoylation (Yanai et al., 2006; Lemarié et al., 2021) or N-myristoylation (Martin et al., 2011; Martin et al., 2014).

Concerning the yield of our procedure, we have previously shown that the WT and MUT HTT 1–588 N-terminal fragment are largely soluble (95% of the total protein), when assessed by Triton-X100 fractionation protocol and that the over-expression of HSPB1 can increase the clearance of the MUT huntingtin fragment (Bonavita et al., 2023).

Historically, measurement of autophagy substrate clearance has represented a gold standard quantitative method to assess the activity of the autophagic activity, which is critical for the turn-over of long-lived, aggregation-prone proteins, such as mutant HTT, which are associated with neurodegenerative diseases. These methods however have their own limitations, such as the possibility of changes in protein interaction profile, intracellular localisation or post-translational modification, ultimately affecting the protein turn-over, rather than having a direct autophagy-dependent effect on protein clearance. Hence, the protocol that we describe here could represent a useful complementary approach to be combined with those available for measuring the clearance of autophagy substrates. The protein oligomerization profiling assay we propose here is highly sensitive and could be indeed deployed in in both wild-type, autophagy-competent and autophagy-null cells in order to directly cross-validate the role exerted by autophagy in inducing any change in the oligomerization/aggregation rate, but also to address any additional contribution of protein synthesis or other protein degradation pathways that can ultimately influence the HTT oligomerization profile and its propensity toward aggregation. To this end, the effect of changes in protein neo-synthesis on HTT oligomerization could be also addressed in Tet on/Tet off inducible cell lines, like it has been previously shown for mutant alpha-synuclein, associated with Parkinson’s disease (Webb et al., 2003; Renna et al., 2011; Renna et al., 2013).

Finally, it is equally important to document some of the potential limitations of the protocol, in addition to the critical steps for achieving reliable results and discussed the interpretation of the data produced by each assay, as described above. For instance, one potential concern with the use of traditional western blotting for the analysis of over-expressed proteins including HTT is the limited signal linearity generated by enhanced chemiluminescence (ECL) detection. Such a limitation might be resolved by using fluorescent-based detection systems, which have a greater linear range, in comparison to standard ECL. These fluorescent-dye conjugated secondary antibodies can be deployed in the same protocol described in this method article and can be analysed with a fluorescent scanner (Odyssey, LI-COR).

We believe that the protocol we have described here represents a robust, reliable and convenient tool to analyse the oligomerization profile of HD-associated mutant HTT protein in mammalian cell-based systems. Furthermore, it is adaptable to the analysis of several other aggregation-prone proteins associated to human diseases, such as mutant alpha-synuclein, Tau, FUS, ZNF and TDP-43 (Menzies et al., 2017).

In addition, this method could help, in combination for instance with a mutational analysis, in identifying novel molecular determinants, which could become possible targets to either reduce aggregation or induce disaggregation of the mutant protein. To this end, the relevant fractions could be subjected to immune-isolation and to targeted proteomic analysis to identify novel components of the HTT-protein complexes. Using a similar approach, the effect of novel genetic or pharmacological modulators of HTT aggregation could be validated by using this method. Such an aspect could be relevant also for the identification of molecular factors involved in HTT aggregation, or in the regulation of the prion-like propagation of the protein observed in HD (Cicchetti et al., 2014; Jeon et al., 2016), which has recently emerged as a common pathological feature in many other neurodegenerative diseases, including AD, PD and several forms of FTDs (Stopschinski and Diamond, 2017).

## Data Availability

The raw data supporting the conclusion of this article will be made available by the authors, without undue reservation.
