# Supplementary material for: A method for the analysis of the oligomerization profile of the Huntington’s disease-associated, aggregation-prone mutant huntingtin protein by isopycnic ultracentrifugation
Source: Front Mol Biosci. 2024 Jun 27;11:1420691. doi: 10.3389/fmolb.2024.1420691 (PMC11236693; doi:10.3389/fmolb.2024.1420691)
Supplement: Supplementary file 1 [file DataSheet1.PDF]

SUPPLEMENTARY INFO

A method for the analysis of the oligomerization profile of the Huntington's disease-associated, aggregation-prone mutant huntingtin protein by isopycnic ultracentrifugation

Raffaella Bonavita, Rosaria Di Martino, Giuseppe Cortone, Antonello Prodomo, Mariagrazia Di Gennaro, Gianluca Scerra, Valentino Panico, Silvia Nuzzo, Marco Salvatore, Sarah V. Williams, Fulvia Vitale, Maria Gabriella Caporaso, Massimo D'Agostino, Francesca M. Pisani, Angeleen Fleming and Maurizio Renna

This file contains:

1. Supplementary Methods
2. Supplementary Results
3. Supplementary Figure S1-S3
4. Supplementary References

1. Size Exclusion Chromatography (SEC) analysis

The analysis by size exclusion chromatography (SEC) of the oligomerization profile of mutant huntingtin N-terminal fragment was performed as previously reported (Arrigo et al., 2018; Mymrikov et al., 2020), with some slight modifications.

HeLa cells were transiently transfected with either WT-HTT or mut-HTT for 48 hours. Cells were washed with ice-cold PBS, scraped off in PBS, and pelleted by centrifugation at 500x g for 5 min. The cells were lysed mechanically in RAB buffer (100mM Tris-HCl pH 7.5, 0.5mM $MgSO_4$, 1mM EGTA, 2mM DTT) supplemented with protease inhibitors mix by passing them 20 times through a 1ml syringe (equipped with a 29G needle), as already detailed in the method section. Cell debris was removed by centrifugation at 16,900*xg* for 15 min at 4°C. Cleared cell lysates (500 ml) contained approximatively 2.5-3.0 mg of total protein extracts, as determined by Bradford assay using the Quick Start™ Bradford Kit 2 (Bio-Rad, cat. No.: # 500-0205).

Size exclusion chromatography (SEC) was performed with the analytic ÄKTA pure™ chromatography System (Cytiva Life Sciences), using a Superdex™ 200 increase 10/300 GL column (GE Healthcare), previously equilibrated in RAB buffer and subjected to a constant flow rate of 0,2ml/min (Sellberg et al., 2017). 30 fractions (volume: 1ml for each fraction) were collected and aliquots from each fraction (volume: 30μL) were solubilised in Laemmli buffer for 30 min on ice and finally analysed by SDS-PAGE and western blot, as already detailed in the method section.

To calculate the MW of the target protein, a column calibration was performed by using the Gel Filtration Protein Standard (Bio-Rad, cat. No.: # 1511901), and in particular the following proteins were used as reference: blue dextran (2000 kDa), thyroglobulin (669 kDa), γ-globulin (158 kDa), ovalbumin (44 kDa) and myoglobulin (17 kDa) (table in Figure S1A). For each calibration standard, the gel-phase distribution coefficient (Kav) was calculated: Kav = (Ve - Vo)/(Vc - Vo), where Ve = elution volume, Vo = column void volume (7.96 mL based on Blue dextran elution volume), Vc = geometric column volume (24 mL). Then, the calibration curve was plotted using the gel-phase distribution coefficient (Kav) of each calibration standard versus the logarithm of its molecular weight (log MW), generating the equation: $\log(MW) = m * Kav + b$, where m is the slope and b is the intercept on the Kav axis (Figure S1A). This equation is used to determine the MW of the target protein based on its elution volume (Ve). (Gantke et al., 2013; O'Fágáin et al., 2011; Yusifov et al., 2010).

2. Size Exclusion Chromatography (SEC) analysis of the 1-588 N-terminal fragment of the wild-type and mutant huntingtin

In order to analyse by size exclusion chromatography (SEC) the oligomerization profile of mutant HTT, HeLa cells were transiently transfected with either the WT or the mutant FLAG/1-588 over-expression construct. Cell extracts were prepared by mechanical homogenization in RAB buffer, cleared by centrifugation and 2.5-3.0 mg of total extracts run through a size exclusion chromatography column. 30 fractions were collected (1ml for each fraction) and the resulting chromogram confirmed the presence of proteins/protein complexes between fractions 6-7 (i.e., high molecular weights) and 26-27 (i.e., low molecular weights) for both experimental conditions (Figure S1B; orange line for WT HTT and blue line for mutant HTT, respectively). Aliquots from each fraction (more specifically, from fraction 5 to 30, as the first four fractions will contain only the flow-through buffer, as it can also be appreciated in Figure S2A and S3A) were then solubilised and analysed by western blot. We were able to detect both WT and MUT HTT (Figure S2B and S3B, respectively). In particular, the WT HTT was detectable between fractions 9 and 14, which range (according to the calibration curve) from 840 kDa to 100 kDa (Figure S2B, blue dotted-line box), whereas the MUT HTT was detectable between fractions 8 and 14, which range from 905 kDa to 100 kDa (Figure S3B, red dotted-line box). In both cases, neither the WT nor the MUT HTT protein was detectable in the lighter fractions (namely, fractions 15-30) collected from the column. Interestingly, along with the monomeric species, upon SEC analysis it was possible to detect for both the WT and MUT HTT protein the presence of likely dimeric or even (homo- or hetero-) multimeric species (please, refer to the relative position of the bands contained within the blue and red right bracket in Figure S2B and S3B, respectively). In addition, from a qualitative standpoint, it was also possible to observe a tendency of the MUT variant (bearing the expanded poly-Q tract), to generate protein complexes with a relatively higher molecular weight, compared to the WT protein (for instance, compare the shifts and the variations in relative ratios between WT and MUT HTT in lanes 8-9, lanes 11-12 or even 13-14). All these data are entirely consistent with the results derived from our ultracentrifugation-based analytical method, as well as with what has been previously reported in the literature (Bäuerlein et al., 2017; Chen et al., 2017; Pandey et al., 2018; Tao et al., 2019).

3. Supplementary Figures

FIGURE S1

A

REFERENCE PROTEIN	*Size (M.W.)*
Myoglobin	17 kDa
Ovalbumin	45 kDa
γ-globulin	158 kDa
Thyroglobulin	669 kDa
Blue dextran	2000 kDa

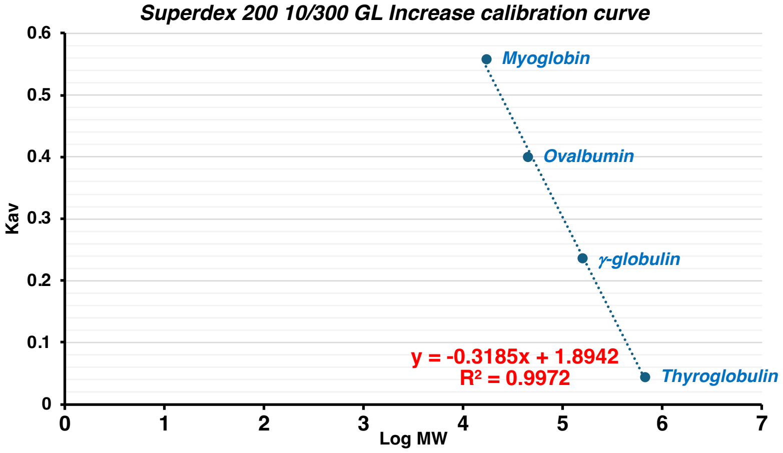

B

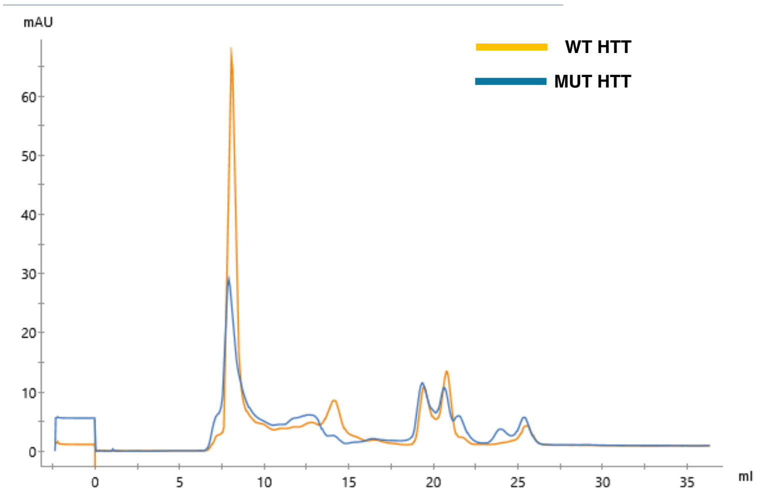

Figure S1: Size Exclusion Chromatography (SEC) analysis of WT and MUT HTT 1-588 N-terminal fragment

(A) To calculate the MW of the target protein a column calibration has been performed. For each calibration standard, the logarithm of its known molecular weight [log(MW)] was plotted against its normalized elution volume (Ve/Vo), generating the equation: log (MW) = m(Ve/Vo) + b, where m is the slope and b is the intercept, as detailed in the method section. The equation is then used to determine the MW of the target protein, based on its elution volume (Ve). **(B)** HeLa cells were transiently transfected with either WT-HTT or mut-HTT for 48 hours. Cells were lysed mechanically in RAB buffer containing protease inhibitor mix and cleared cell lysates (500 ml) containing about 2.5 mg of total protein extracts were subjected to size exclusion chromatography (SEC) with the analytic ÄKTA System, using a Superdex™ 200 increase 10/300 GL column at a flow rate of 0,2ml/min. 30 fractions (volume: 1ml) were collected as reported in the chromogram, which confirmed the presence of proteins/protein complexes between fractions 6-7 (i.e., high molecular weights) and 26-27 (i.e., low molecular weights) for both conditions (orange line for WT HTT and blue line for MUT HTT, respectively).

FIGURE S2

A

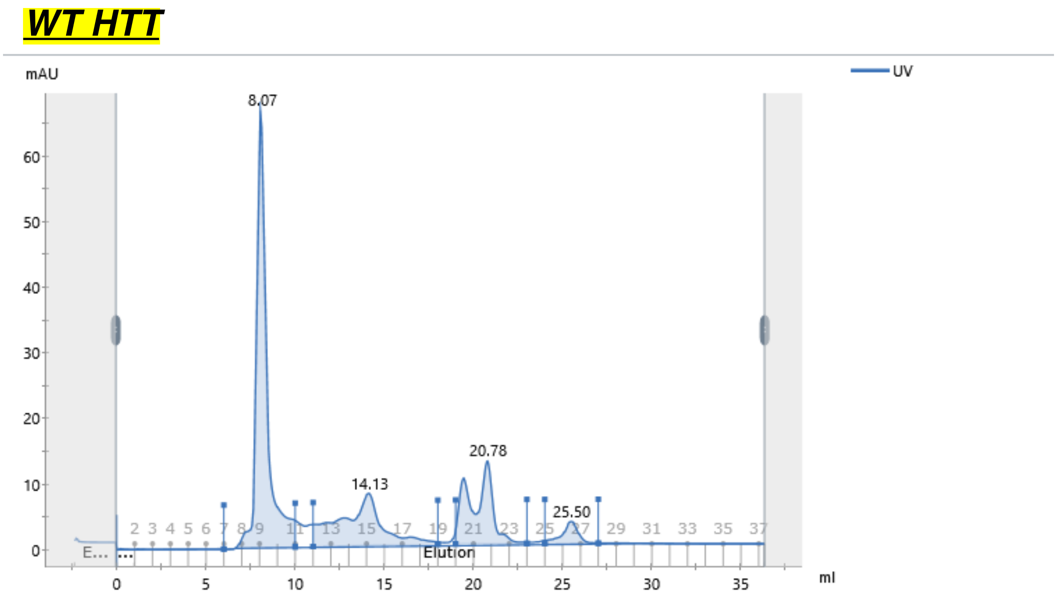

B

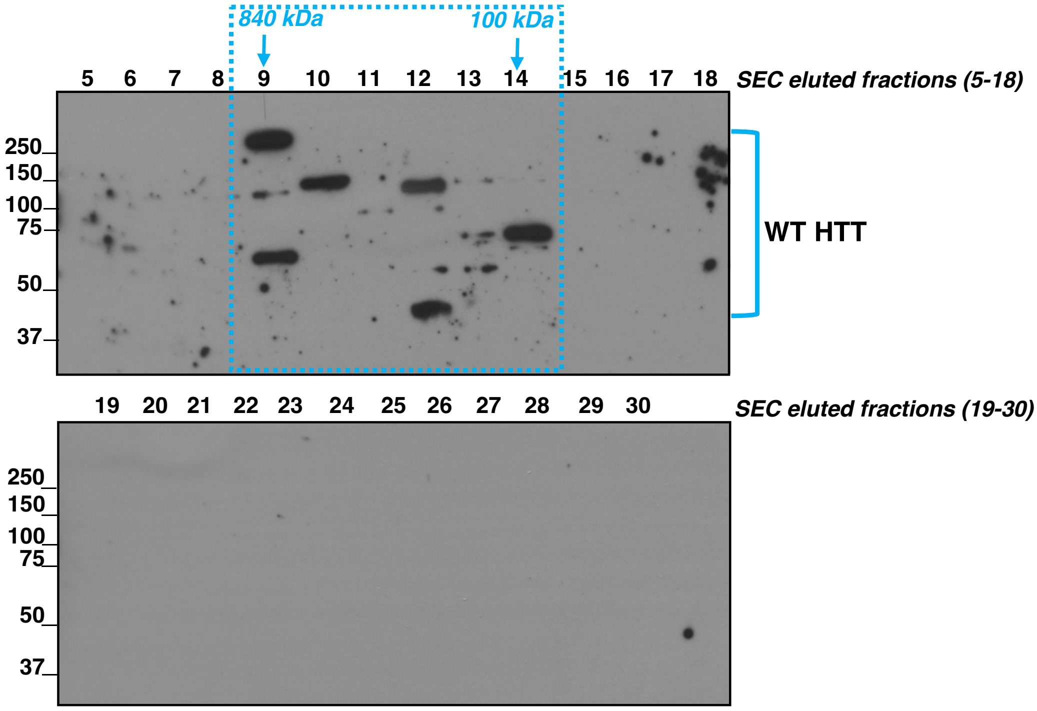

Figure S2: Size Exclusion Chromatography (SEC) analysis of WT HTT N-terminal fragment **(A)** HeLa cells were transiently transfected with WT-HTT for 48 hours. Cells were lysed mechanically in RAB buffer and 2.5 mg of total protein extracts were subjected to size exclusion chromatography (SEC) with the analytic ÄKTA System, using a Superdex™ 200 increase 10/300 GL column, previously equilibrated in RAB buffer at a flow rate of 0,2ml/min. 30 fractions (volume: 1ml) were collected as reported in the chromogram, which confirmed the presence of proteins/protein complexes between fractions 6-7 (i.e., high molecular weights) and 26-27 (i.e., low molecular weights). Then, the equation is used to determine the MW of the target protein based on its elution volume (Ve), which is indicated at the top of each peak. **(B)** Aliquots from fraction 5 to 30, were solubilised and analysed by Western blot analysis. The WT HTT was detectable between fractions 9 and 14, which range from 840 kDa to 100 kDa (blue dotted-line box). Neither the WT nor the MUT protein was detectable in the lighter fractions (15-30) collected from the column.

FIGURE S3

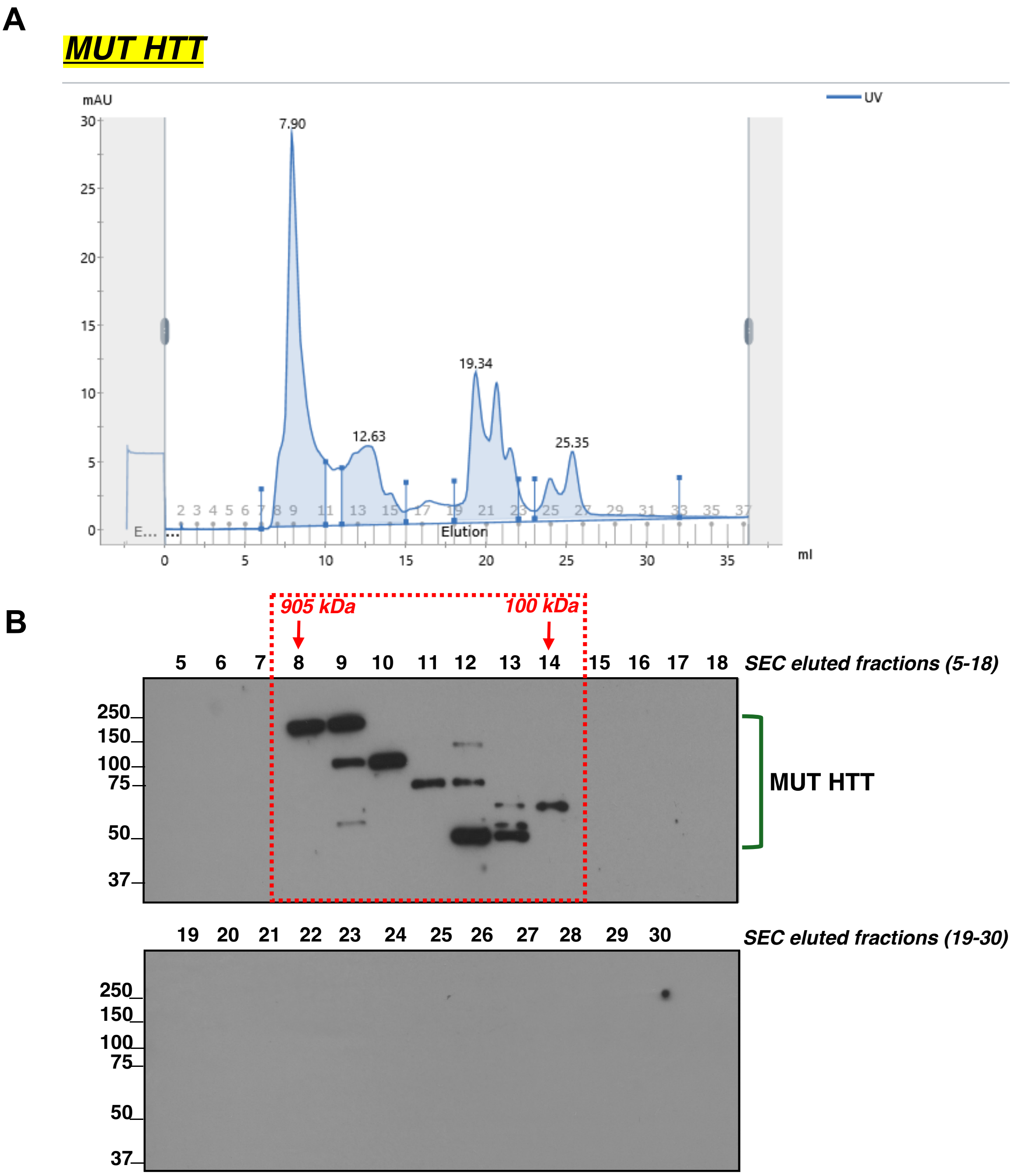

FIGURE S3 Size Exclusion Chromatography (SEC) analysis of MUT HTT N-terminal fragment (A) HeLa cells were transiently transfected with MUT HTT for 48 hours. Cells were lysed mechanically in RAB buffer and 2.5 mg of total protein extracts extracts were subjected to size exclusion chromatography (SEC) with the analytic ÄKTA System, using a Superdex™ 200 increase 10/300 GL column. 30 fractions (volume: 1ml) were collected as reported in the chromogram (A), which confirmed the presence of proteins/protein complexes between fractions 6-7 (i.e., high molecular weights) and 26-27 (i.e., low molecular weights). The elution volume (Ve) is indicated at the top of each peak. **(B)** Aliquots from fraction 5 to 30 were solubilised and analysed by Western blot analysis. The MUT HTT was detectable between fractions 8 and 14, which range from 905 kDa to 100 kDa (red dotted-line box). Neither the WT nor the MUT protein was detectable in the lighter fractions (15-30) collected from the column.

4. Supplementary References

Arrigo, A.P. (2018). Analysis of HspB1 (Hsp27) Oligomerization and Phosphorylation Patterns and Its Interaction with Specific Client Polypeptides. Calderwood, S., Prince, T. (eds) Chaperones. Methods in Molecular Biology, vol 1709. Humana Press, New York, NY. DOI: 10.1007/978-1-4939-7477-1 12

Bäuerlein, F.J.B., Saha, I., Mishra, A., Kalemanov, M., Martinéz-Sánchez, A., Klein, R., Dudanova, I., Hipp, M.S., Hartl, F.U., Baumeister, W., Fernández-Busnadiego, R. (2017). In situ architecture and cellular interactions of poly-Q inclusions. *Cell.* 171,179-187.

Chen, M., Wolynes, P.G. (2017). Aggregation landscapes of huntingtin exon 1 protein fragments and the critical repeat length for the onset of Huntington's disease. *Proc Natl Acad Sci USA* 114, 4406-4411. DOI: 10.1073/pnas.1702237114

Gantke, T., Boussouf, S., Janzen, J., Morrice, N.A., Howell, S., Mühlberger, E., Ley S.C. (2013). Ebola virus VP35 induces high-level production of recombinant TPL-2-ABIN-2-NF-κB1 p105 complex in co-transfected HEK-293 cells. Biochem. J. 452(2): 359-365. DOI: 10.1042/BJ20121873.

Mymrikov, E.V., Riedl, M., Peters, C., Weinkauf, S., Haslbeck, M., Buchner J. (2020). Regulation of small heat-shock proteins by hetero-oligomer formation. *J. Biol. Chem.* 295(1) 158-169 DOI: 10.1074/jbc.RA119.011143

O'Fágáin C., Cummins, P.M., O'Connor, B.F. (2011). Gel-filtration chromatography. Methods Mol. Biol. 681:25-33. DOI: 10.1007/978-1-60761-913-0_2.

Pandey, N.K., Isas, J.M., Rawat, A., Lee, R.V., Langen, J., Pandey, P., and Langen, R. (2018). The 17-residue-long N terminus in huntingtin controls stepwise aggregation in solution and on membranes via different mechanisms. *J. Biol. Chem.* 293, 2597-2605.

Sellberg, A., Holmqvist, A., Magnusson, F., Andersson, C., Nilsson, B. (2017). Discretized multi-level elution trajectory: A proof-of-concept demonstration. J. Chromatogr. A. 1481: 73-81. DOI: 10.1016/j.chroma.2016.12.038.

Tao, M., Pandey, N.K., Barnes, R., Han, S., Langen, R. (2019). Structure of Membrane-Bound Huntingtin Exon 1 Reveals Membrane Interaction and Aggregation Mechanisms. *Structure.* 27, 1570-1580. DOI: 10.1016/j.str.2019.08.003.

Yusifov, T., Javaherian, A.D., Pantazis, A., Gandhi, C.S., Olcese R. (2010). The RCK1 domain of the human BKCa channel transduces Ca2+ binding into structural rearrangements. J. Gen. Physiol. 136(2): 189-202. DOI: 10.1085/jgp.200910374
